# Widespread 3′-end uridylation in eukaryotic RNA viruses

**DOI:** 10.1038/srep25454

**Published:** 2016-05-06

**Authors:** Yayun Huo, Jianguo Shen, Huanian Wu, Chao Zhang, Lihua Guo, Jinguang Yang, Weimin Li

**Affiliations:** 1 Biotechnology Research Institute, Chinese Academy of Agricultural Sciences, Beijing, China; 2 Inspection & Quarantine Technology Center, Fujian Entry-Exit Inspection and Quarantine Bureau, Fuzhou, China; 3 Institute of Plant Protection, Chinese Academy of Agricultural Sciences, Beijing, China; 4 Tobacco Research Institute, Chinese Academy of Agricultural Sciences, Qingdao, China

## Abstract

RNA 3′ uridylation occurs pervasively in eukaryotes, but is poorly characterized in viruses. In this study, we demonstrate that a broad array of RNA viruses, including mycoviruses, plant viruses and animal viruses, possess a novel population of RNA species bearing nontemplated oligo(U) or (U)-rich tails, suggesting widespread 3′ uridylation in eukaryotic viruses. Given the biological relevance of 3′ uridylation to eukaryotic RNA degradation, we propose a conserved but as-yet-unknown mechanism in virus-host interaction.

Addition of non-templated nucleotides to 3′ -ends of RNA transcripts is a well-known type of post-transcriptional RNA modification in kingdoms of life. The most understood example within this category is polyadenylation, by which multiple adenosines are progressively added to the 3′ -ends of RNA substrates by the canonical poly(A) polymerase (PAP) or non-canonical PAP, thereby resulting in poly(A) or poly(A)-rich tails^1^,^2^,^3^. This process occurs in almost all organisms but plays opposite roles in control of RNA stability. The long poly(A) tail at the mature 3′ -ends of nucleus-encoded mRNAs in eukaryotes is a key determinant of transcripts stability, as well as nucleocytoplasmic export and translation initiation^1^,^4^. By contrast, the poly(A) or poly(A)-rich stretches, which are associated with the fragmented molecules of both coding and non-coding RNAs in prokaryotes, eukaryotes and organelles, serve as toeholds for 3′ to 5′ exoribonucleases to attack the RNA^2^,^5^,^6^.

Along with polyadenylation, uridylation is another important type of RNA tailing, and has been recently observed in various eukaryotes, from fission yeast to human^7^,^8^,^9^. Many classes of RNA species, such as U6 snRNA, mRNAs, small RNAs and RNA-induced silencing complex (RISC)-cleaved fragments, are subjected to 3′ uridylation by the enzymes referred to as terminal uridyltransferases or poly(U) polymerase (PUP), which are in fact some non-canonical PAPs with capacity to catalyze uridylation instead of adenylation^8^,^10^,^11^,^12^. As far as is known, 3′ uridylation can contribute function through RNA editing, as showed in the mitochondria of trypanosomes and *Leishmania*^13^, and also play roles in microRNA (miRNA) biogenesis, as in the case of let-7 miRNA in human cells^14^. Additionally, like polyadenylation, the non-templated 3′ uridines also confer opposite roles on RNA stability. The stabilizing effect has been discovered in U6 snRNA, whose 3′ -end is stabilized after post-transcriptional addition of uridines followed by formation of cyclic 2, 3-phosphate at the final nucleotide^15^. On the contrary, 3′ uridine added to mRNAs as well as 5′ mRNA fragments formed by the RISC cleavage enhances degradation of these types of RNA species but with somewhat different mechanisms. For instance, 3′ uridylation promotes histone mRNA degradation by decapping and simultaneous exonucleolytic digestion of the mRNA both 3′ to 5′ and 5′ to 3′ 16. The 3′ U-tract addition at oligoadenylated mRNAs and 5′ products from the RISC cleavage, however, protects RNA from 3′ → 5′ exonucleolytic decay but stimulates decapping followed by 5′ → 3′ degradation, thus favoring RNA decay solely in the 5′ → 3′ direction^12^,^17^,^18^,^19^. Uridylation also triggers decay of the mature miRNAs and siRNAs (small interfering RNA) in *C. elegans*^20^, *C. reinhardtii*^21^, zebrafish^22^ or Arabidopsis^23^,^24^ as well as the let-7 miRNA precursors in *C. elegans*^25^ or human embryonic and cancer cells^26^,^27^,^28^. To date, the directionality of small RNAs decay remains unclear but probably achieves in 3′ → 5′ , given the fact that 3′ -terminal methylation of miRNAs and siRNAs is able to block 3′ uridylation and thus stabilize the small RNAs^29^.

Viruses are not included in kingdoms of life, but are small infectious agents which can replicate only within the living cells of organisms. It has long been known that RNAs of many DNA and RNA viruses in eukaryotes undergo polyadenylation, thereby generating 3′ poly(A) tails to maintain RNA stability as well as initiate translation^30^,^31^,^32^. Recently, the 3′ poly(A) or 3′ poly(A)-rich moieties probably conferring RNA instability were detected in a broad range of positive-strand RNA plant viruses as well as a double-stranded RNA plant virus known to lack poly(A) tail^33^,^34^, indicating that occurrence of RNA polyadenylation in viruses is much more common than we currently appreciate.

In contrast to the familiar 3′ polyadenylation, RNA 3′ uridylation of viruses is poorly characterized. However, a few studies on *Beet necrotic yellow vein virus* (BNYVV), Sindbis virus (SIN), coxsackievirus B3 (CVB3) and hepatitis C virus (HCV) once disclosed that after removal of the 3′ poly(A) tails from genomes of these four polyadenylated positive-strand RNA viruses, their progeny would regain a 3′ tail that contain not just a poly(A) tail but also a U-rich or AU-rich linker preceding the poly(A)^35^,^36^,^37^,^38^. A similar observation was also made on a DNA virus, Epstein-Barr virus (EBV). Sequencing of a truncated EBV *pol* mRNA cleaved by a virus-encoded miRNA identified a non-templated AU-rich region followed by a poly(A) tail^39^,^40^. While the mechanism that generates the U-rich or AU-rich tract in viral RNAs and its significance remain undetermined yet^32^,^41^, the bodies of evidence suggest that many, if not all viruses, do bear RNA uridylation.

To determine the extent of RNA 3′ uridylation in viruses, herein we examined a broad range of RNA viruses infecting either lower eukaryotes (fungi) or higher eukaryotes (plants and animals). By sequencing 3′ -termini of the viral RNAs, we show that, although belonging to phylogenetically distinct groups, none of the tested RNA eukaryotic viruses is free of 3′ uridylation. The data unambiguously demonstrated the widespread 3′ uridylation in eukaryotic RNA viruses, suggesting that viral RNA 3′ uridylation is conserved across eukaryotes and may play an unknown role in host and virus interaction.

## Results and Discussion

**Figure 1:**
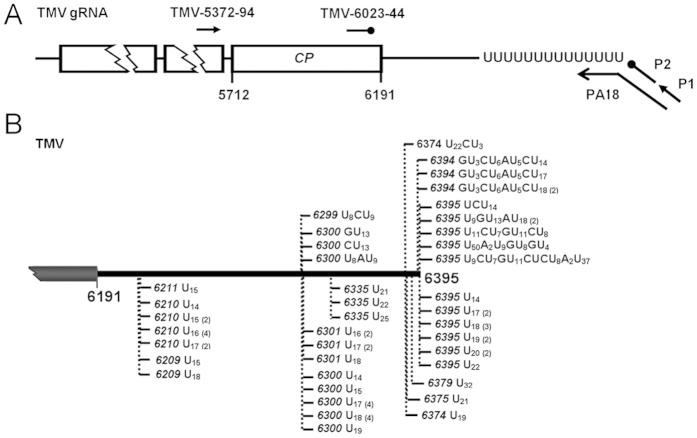
Identification of the TMV RNA species bearing 3′ uridine tails. (**A**) Schematic diagram of the oligo(dA) primed RT-PCR. The primers corresponding to the TMV genome were listed in Supplementary Table 1. (**B**) Nature of 3′ uridine tails associated with TMV RNAs. The 3′ end of TMV genome is schematically diagramed. Tails are detected from the *N. benthamiana* leaves inoculated with TMV at 4 dpi. Nucleotide compositions of the 3′ tails are shown, and vertical dashed lines with numbers indicate positions of the tails on TMV genome.

Following the previous evidence that non-templated 3′ uridine addition takes place in BNYVV, SIN, CVB3, HCV and EBV, the viral genomic RNAs or mRNAs of which all bear 3′ poly(A) tails^35^,^36^,^37^,^38^, we questioned whether RNA 3′ uridylation occurs only in viruses with polyadenylated genomic RNA/mRNA. To address this concern, an initial test was performed on *Tobacco mosaic virus* (TMV, *Tobamovirus*, *Virgaviridae*), one distinguished RNA plant virus whose genome terminates with tRNA-like structures (TLS)^42^. A simple approach of oligo(dA) primed RT-PCR (Fig. 1A), which is modified from the oligo(dT) primed RT-PCR^33^,^43^, was used to characterize the 3′ end of viral genomes. In brief, total RNA of the TMV-inoculated *Nicotiana benthamiana* leaves was first reverse transcribed with an anchored oligo(dA) primer PA18 followed by a nested PCR with the primer pair of P1/TMV-5372-94 and P2/TMV-6023-44 (Fig. 1A and Supplementary Table 1). The resulting PCR products were then cloned and sequenced. By this approach, we successfully isolated the TMV RNA species carrying non-templated uridines at their 3′ ends (Fig. 1B). Of note, we have recently characterized a number of TMV RNAs bearing 3′ poly(A) or poly(A)-rich tails, wherein nonetheless lie no any apparent U or U-rich region inside. Therefore, the uridine sequences of TMV RNAs detected here should not be internal architectures preceding the poly(A) tails as observed in BNYVV, SIN, CVB3, HCV and EBV^35^,^36^,^37^,^38^, but were of 3′ tail indeed. Additionally, to ensure that the 3′ uridine tails of TMV RNAs were not amplification artifacts, we further examined a RNA mixture containing 0.1 μ g *in vitro* TMV RNA transcripts known to lacking oligo(U) tails and 0.9 μ g total RNA from healthy *N. benthamiana* leaves with the same approach. As a result, no viral RNA with 3′ uridine tail was cloned (data not shown), thus confirming 3′ uridylation of TMV RNAs.

To better understand structures of the 3′ uridine tails added to viral RNAs, we totally cloned and analyzed 60 uridylated TMV RNA molecules. Two types of tails were identified, including 45 oligo(U) tails comprised exclusively of uridines, and 15 oligo(U)-rich tails that contain mostly uridines (96.77%) with a few guanosines (0.9%), adenosines (0.9%) and cytosines (1.43%) interspersed. In particular, the oligo(U) tails ranged from 14 nt to 32 nt with a mean size of 17.87 nt, whereas the oligo(U)-rich tails averaged up to 34.93 nt with a significant variation from 14 nt to 79 nt. However, it is worthy to emphasize that true length of the oligo(U) and oligo(U)-rich tails added to TMV RNAs is probably longer than those we detected, regarding that the oligo(dA)-dependent RT-PCR used here does not allow properly scaling the full length of the 3′ uridine tails, as the limitation of the oligo(dT)-dependent RT-PCR discussed previously^33^,^43^. Nonetheless, these data provided basic information of 3′ uridine tails in TMV RNAs, and denoted a preference for UTP over ATP, CTP and GTP in the process of the 3′ oligo tails synthesis.

A particular interest was also given to the junction sites between the TMV RNAs and non-templated 3′ uridine tails. By thoroughly dissecting the collected TMV RNA-oligo(U) species, we identified that 51 out of 60 tails were appended to nts 6209–6211, 6299–6301 and 6394–6395 of TMV genome, which were assumed as hotspots for uridine addition. The data also pinpointed that a large majority of cloned tails were located at positions ranging from 1 to 175 nt upstream of the mature 3′ -end of TMV genome, whereas only 17 tails directly added to the viral RNAs bearing intact 3′ -end, suggesting the viral RNAs with incomplete 3′ -ends, in comparison with the full length TMV genomes, were more likely targeted for 3′ uridylation. In addition, we noted that the 3′ terminal-nucleotides of the TMV RNAs adjunct with uridine tails were mostly adenosines and uridines (> 61.7%), as observed in the TMV RNA species carrying poly(A) or poly(A)-rich tails^33^. Referring to the role of ncPAPs in RNA tailing^44^, the data herein implied that the ncPAP(s) in *N. benthamiana* might tend to uridylate the TMV RNA species ended with adenosine or uridine.

Presence of 3′ uridine tails in TMV RNAs prompted us to explore if RNA 3′ uridylation generally occurs in positive-strand RNA viruses, the largest class within the eukaryotic RNA viruses^30^. It is known that positive-strand RNA viral genome, similar to mRNA, can be directly translated by host ribosomes for protein synthesis. Their 3′ -terminal structures, however, are variable and contain not just poly(A) tail but TLS and non-TLS heteropolymeric sequence (Het)^31^. Additional examinations were then extended to nine more positive-strand RNA viruses which harbor diverse 3′ -terminal structures. For instance, CMV (*Cucumber mosaic virus*, *Cucumovirus*, *Bromoviridae*), TRV (*Tobacco rattle virus*, *Tobravirus*, *Virgaviridae*) and ORSV (*Odontoglossum ringspot virus*, *Tobamovirus*, *Virgaviridae*) terminate with 3′ TLS, PVX (*Potato virus X*, *Potexvirus*, *Alphaflexiviridae*), PVY (*Potato virus Y*, *Potyvirus*, *Potyviridae*), PEDV (Porcine epidemic diarrhea virus, *Alphacoronavirus*, *Coronaviridae*), PRRSV (Porcine respiratory and reproductive syndrome virus, *Arterivirus*, *Arteriviridae*) and FgHV2 (Fusarium graminearum hypovirus 2, *Hypovirus*, *Hypoviridae*) bear 3′ poly(A) tail, and TCV (*Turnip crinkle virus*, *Carmovirus*, *Tombusviridae*) has 3′ Het. As is known, FgHV2 belongs to mycovirus, PEDV and PRRSV are animal viruses, and CMV, TRV, ORSV, PVX, PVY and TCV infect plants. Hosts of these viruses range from lower eukaryotes to higher eukaryotes, presenting a vast phylogenetic diversity.

**Figure 2:**
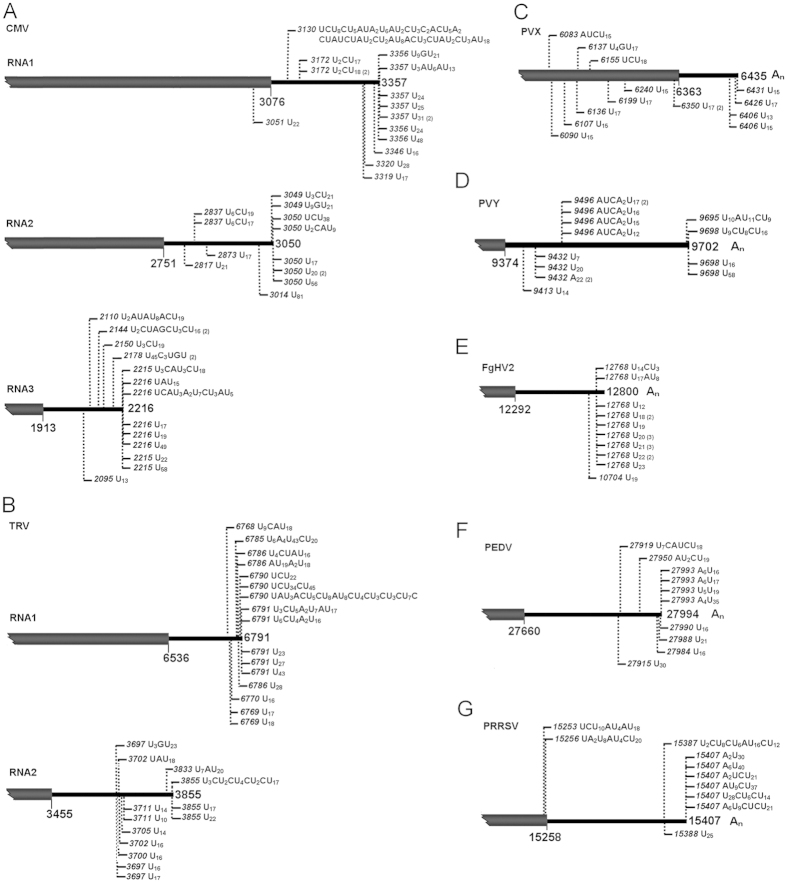
The 3′ uridine tails are present in diverse positive-strand RNA viruses. The tails associated with viral RNAs were detected from the CMV-, TRV-, PVX- and PVY-inoculated *N. benthamiana* leaves, the FgHV2-infected *F. graminearum*, the PEDV-infected Vero cells, or the PRRSV-infected MARC-145 cells (**A–G**). Nucleotide compositions of the 3′ tails are shown, and vertical dashed lines with numbers indicate positions of the tails on viral genomes.

**Figure 3:**
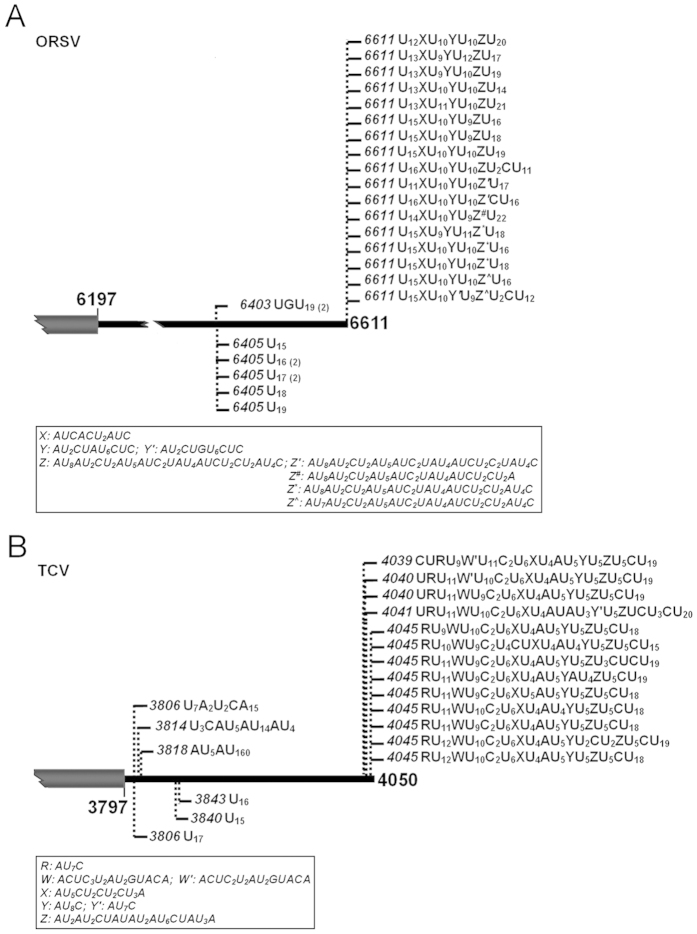
The 3′ uridine tails of ORSV and TCV bear consensus elements. Tails were isolated from the ORSV (**A**) or TCV (**B**) inoculated *N. benthamiana* leaves. Nucleotide compositions of the 3′ tails are shown, and vertical dashed lines with numbers indicate positions of the tails on viral genomes. The consensus elements of ORSV and TCV were shown in the box, respectively.

The same approach of oligo(dA) primed RT-PCR was used to scrutinize the putative uridylated viral RNA species from total RNA of the FgHV2-infected *Fusarium graminearum*, the PEDV-infected Vero cells, the PRRSV-infected MARC-145 cells, and the *Nicotiana benthamiana* leaves inoculated with CMV, TRV, ORSV, PVX, PVY and TCV, respectively. With the corresponding primer pairs (Supplementary Table 1), we identified that all the tested viruses, which represent any of the three typical 3′ terminal structures, have RNA species bearing 3′ tails of oligo(U) or oligo(U)-rich but with somewhat differences (Figs 2 and 3). With respect to locations of the 3′ uridine tails, the ones cloned from CMV (RNA1, RNA2 and RNA3), TRV (RNA1 and RNA2), PRRSV and ORSV were at and upstream of 3′ end of genomic RNA (Figs 2A,B,G and 3A), like those of TMV (Fig. 1B). By contrast, no tail from PVX, PVY, FgHV2, PEDV and TCV was located at the mature 3′ end of viral genome (Figs 2C–F and 3B). In particular, 14 out of 16 uridine tails associated with the FgHV2 RNA are exclusively composed of uridines (Fig. 2E), suggesting the preferential homopolymeric uridylation. Notably, in contrast to the low similarities among the oligo(U)-rich tails identified from any other tested viruses, the tails at or near the mature 3′ end of viral genomes of ORSV and TCV shared nucleotide sequences with quite a few consensus entities, as shown in Fig. 3A,B. This is reminiscent of a previous study on fungus *Aspergillus nidulans*, in which the non-templated 3′ tails associated with the deadenylated mRNAs also bear consensus element like CUCU, and are generated through a nucleotidyltransferase namely cutA^45^. Herein, the 3′ tails with consensus were present in only ORSV and TCV but not all positive-strand RNA viruses propagated in *N. benthamiana*, implying that a nucleotidyltransferase like cutA might lie in this plant species and have an ability to selectively recognize the RNAs to form the unusual kind of 3′ uridine tails.

Thus, the data unambiguously demonstrated presence of the 3′ uridine tails in positive-strand RNA eukaryotic viruses varied with 3′ terminal structure [Poly(A), TLS and Het] as well as host range (fungi, plants and animals). Regarding prevalence of 3′ uridylation in positive-strand RNA viruses, we next investigated uridylation in another type of single-stranded RNA viruses, negative-strand RNA viruses.

**Figure 4:**
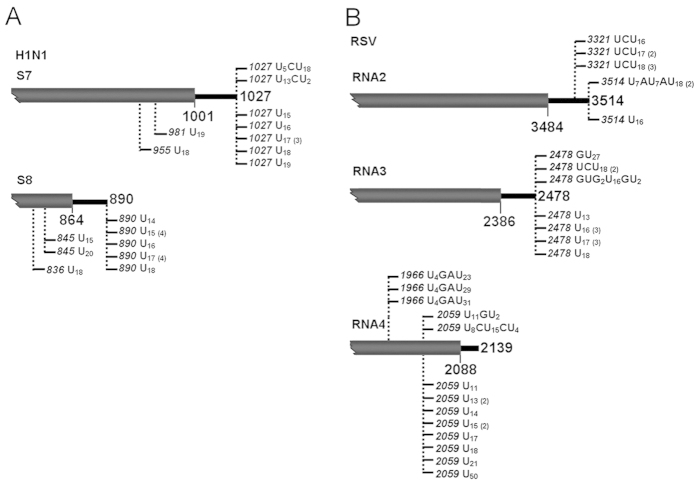
The 3′ uridine tails are present in negative-strand RNA viruses. Tails associated with viral RNAs corresponding to H1N1 segments 7 and 8 (**A**) as well as RSV segments 2, 3 and 4 (**B**) were detected from the RSV-infected rice leaves and the H1N1-infected MDCK cells. Nucleotide compositions of the 3′ tails are shown, and vertical dashed lines with numbers indicate positions of the tails on viral genomes.

To this end, Influenza A (H1N1) virus (*Influenzavirus A*, *Orthomyxoviridae*) and *Rice stripe virus* (RSV, *Tenuivirus*, unassigned family), two viruses with segmented negative-sense RNA genomes were examined. By the oligo(dA) primed RT-PCR with specific primers (Supplementary Table 1), total RNA of H1N1-infected MDCK cells and RSV-infected rice leaves was analyzed to dissect 3′ -ends of the putative uridylated RNAs derived from H1N1 segments 7 and 8 as well as RSV segments 2, 3 and 4. In line with the observation from the positive strand RNA viruses, the 3′ uridine tails were successfully identified from the tested viral RNAs of H1N1 and RSV (Fig. 4). Considering nature of the isolated tails, the homopolymeric tails are predominant in H1N1 viral RNAs, in particular segment 8 (Fig. 4A). By contrast, both homo- and hetero-tails associated with RSV segments 2, 3 and 4 (Fig. 4B). Addition sites of the uridine tails are also varied among the different viral RNAs. As shown in Fig. 4A, the tails added to H1N1 segments 7 and 8 and RSV segment 2 were located either at or upstream of the 3′ end of viral RNA. However, the tails with RSV segment 3 were uniquely at the mature 3′ end, and the tails with RSV segment 4 were instead at least 80 nt away from the 3′ terminus (Fig. 4B).

**Figure 5:**
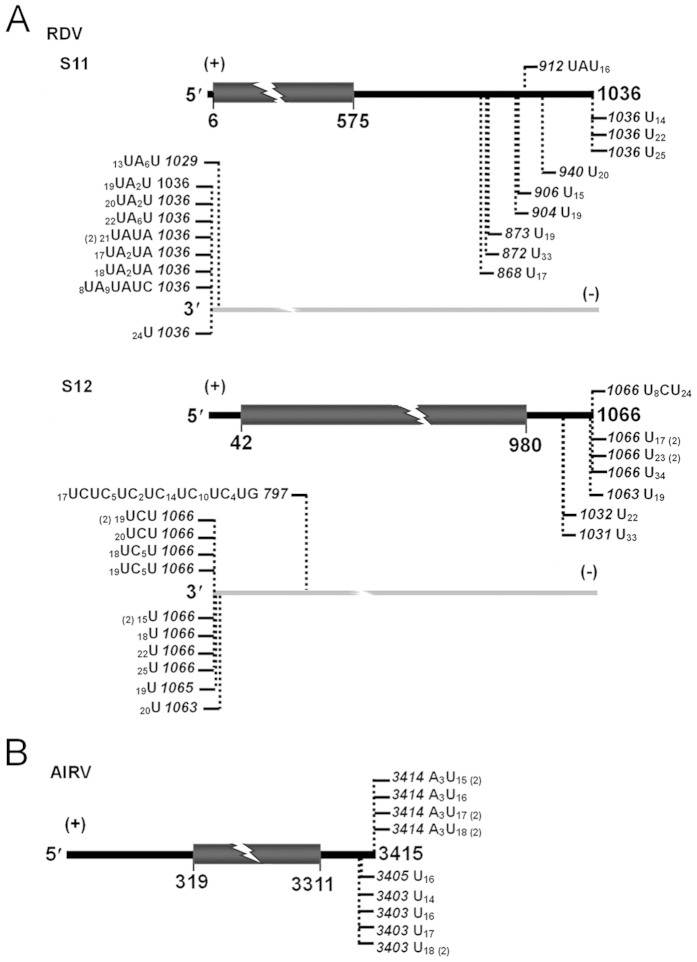
The 3′ uridine tails are present in double-stranded RNA viruses. Tails associated with viral RNAs corresponding to plus and minus strands of RDV segments 11 and 12 (**A**) as well as plus strand of AlRV1 genome (**B**) were detected from the RDV-infected rice leaves and the AlRV1-infected *A. longipes*. Nucleotide compositions of the 3′ tails are shown, and vertical dashed lines with numbers indicate positions of the tails on viral genomes.

Discovery of 3′ uridine tails in viruses with either positive-sense or negative-sense RNA genome was indicative of the wide occurrence of uridylation on single-stranded RNA viruses. Accordingly, we asked if double-stranded RNA eukaryotic viruses are subjected to RNA 3′ uridylation. To address this concern, *Rice dwarf virus* (RDV, *Phytoreovirus*, *Reoviridae*), a typical double-stranded RNA plant virus containing 12 dsRNA segments, was tested. Using total RNA of RDV-infected rice leaves as template, we analyzed both plus and minus strands of RDV segments 11 and 12 with oligo(dA) primed RT-PCR. The result demonstrated that all the tested RNA strands were subjected to 3′ uridylation (Fig. 5A). Interestingly, the tails associated with plus strands of segments 11 and 12 are almost exclusively composed of uridines, whereas their minus strands, in particular the ones of segment 11, exhibited a significant number of oligo(U)-rich tail. Since the plus and minus strands are complementary to each other, the genomes of double-stranded RNA viruses render an ideal model to investigate effect of RNA substrates on UTP selectivity during 3′ uridylation. With the same strategy, we further examined *Alternaria longipes* dsRNA virus 1 (AlRV1), one unclassified dsRNA mycovirus containing a single genome. Consistently, 3′ uridine tails were also present in the plus strand of AlRV1 dsRNAs (Fig. 5B). Such tails, however, were failed to be identified from AlRV1 RNAs in minus sense (data not shown), implying that little or no minus strands of AlRV1 RNAs were uridylated. Recently, the non-canonical 3′ ployadenylation has been defined in a double-stranded RNA virus, *Southern rice black-streaked dwarf virus* (*SRBSDV*, *Fijivirus*, *Reoviridae*)^34^. The data available here supported that another manner of the post-transcriptional modification, RNA 3′ uridylation, occurred also in double-stranded RNA viruses.

In summary, this study characterized a novel type of viral RNA species bearing 3′ uridine tails from positive strand, negative strand or double-stranded RNA viruses with hosts ranging from fungi to plants and animal. Although the impacts of the oligo(U) and (U)-rich tails on viral RNAs remain unknown yet, the tails strictly resemble the previously determined degradation-stimulating oligo(U) tails associated with eukaryotic RNAs^12^,^18^,^44^, implying a novel and conserved mechanism in virus-host interaction. In recent years, increasing evidence indicates that viruses have developed ways of interfacing with the cellular RNA decay machinery in order to stabilize viral transcripts and promote productive infections^46^,^47^,^48^,^49^,^50^. Herein, diverse virus-host systems were identified to generate 3′ uridylated viral RNAs, suggesting that the viruses have probably evolved to circumvent or adapt to the uridylation-stimulated RNA decay mechanism, and thus establish successful infection in their hosts. Nonetheless, our findings disclosed the widespread 3′ uridylation in eukaryotic RNA viruses, and the biological relevance as well as biogenesis of the 3′ uridylated viral RNAs merits future investigation.

## Materials and Methods

### RNA plant viruses and host plants

A total of nine positive strand RNA viruses, TMV, ORSV, CGMMV, CMV, TRV, TCV, TNV, PVX and PVY, were mechanically inoculated in 4-week-old *N. benthamiana* plants cultured at 25 °C with 16h light/8 h dark cycle, following collection of the inoculated leaves at 3 dpi as described^33^. The negative strand RNA virus RSV and the double-stranded RNA virus RDV, however, were inoculated onto 4-week-old rice (*O. sativa* spp. *japonica*) seedlings grown at 28~30 °C under natural sunlight through the viruliferous (RSV and RDV-carrying) insects of *L. striatellus* and *N. cincticeps*, respectively^51^,^52^. The newly developed leaves showing viral symptoms were harvested after ~3 weeks post inoculation.

### RNA animal viruses and mammal cells

Two positive strand RNA viruses, PRRSV HB-1/3.9^53^ and PEDV^54^, and a negative strand RNA virus influenza A/Puerto Rico/8/34 (H1N1, PR8) were used in this study. Cells of MARC-145, VERO CCB-81 and MDCK were maintained in Dulbecco’s modified Eagle’s medium (Invitrogen, USA) supplemented with 10% fetal bovine serum (Hyclone Laboratories Inc., USA) at 37 °C, and were infected with PRRSV, PEDV and H1N1 at multiplicity of infection (MOI) of 0.1, respectively. At 16 post infection (hpi), the supernatant was removed and cells were collected.

### Mycovirus and Fungi

Mycelial plugs of *Fusarium graminearum* strain JS16 with the positive strand RNA virus FgHV2^55^ and *Alternaria longipes* strain HN28 with the double-stranded RNA virus AlRV1^56^ were incubated on Potato Dextrose Agar in Petri plates overlaid with cell ophanemembranes at 25 °C in the dark. The mycelial mass was harvested after 4 days incubation.

### RNA preparation and oligo(dA) primed RT-PCR

Total RNA was extracted from the virus-infected plant leaves, mammal cells or fungi by using TRIzol reagent (Invitrogen, USA) following the manufacture’s protocol. To clone the 3′ uridine tails associated with the viral RNAs, the approach of oligo(dA) primed RT-PCR was designed according to the oligo(dT) primed RT-PCR previously described^33^,^43^. In brief, 1 μ g total RNA was first reverse transcribed by M-MLV reverse transcriptase (Promega, USA) with anchored oligo(dA)_18_ PA18 (Supplementary Table 1), and the resulted first strand cDNA was subsequently used as template to perform the 1^st^ round of PCR amplification with P1 and outer primer (Supplementary Table 1). After the 2^nd^ round of PCR, which was based on the 1^st^ PCR round products as templates, the PCR products obtained by using P2 and inner primer (Supplementary Table 1) were treated with QIAquick PCR Purification Kit (Qiagen, USA) and ligated with pGEM-T easy vector (Promega, USA). Following transformation, the positive clones were randomly selected for sequence analysis.

## Additional Information

**How to cite this article**: Huo, Y. *et al.* Widespread 3′-end uridylation in eukaryotic RNA viruses. *Sci. Rep.* **6**, 25454; doi: 10.1038/srep25454 (2016).

## Supplementary Material

Supplementary Information
