# Supplementary material for: Widespread 3′-end uridylation in eukaryotic RNA viruses
Source: Sci Rep. 2016 May 6;6:25454. doi: 10.1038/srep25454 (PMC4858684; doi:10.1038/srep25454)
Supplement: Supplementary Information [file srep25454-s1.doc]

**TITLE:** Widespread 3′-end uridylation in eukaryotic RNA viruses

**AUTHORS:** Yayun Huo^1^, Jianguo Shen^2^, Huanian Wu^1^, Chao Zhang^1^, Lihua Guo^3^, Jinguang Yang^4^, Weimin Li^1^

**AFFILIATIONS:**

^1^Biotechnology Research Institute, Chinese Academy of Agricultural Sciences, Beijing, China;

^2^Inspection & Quarantine Technology Center, Fujian Entry-Exit Inspection and Quarantine Bureau, Fuzhou, China;

^3^Institute of Plant Protection, Chinese Academy of Agricultural Science, Beijing, China

^4^Tobacco Research Institute, Chinese Academy of Agricultural Sciences, Qingdao, China.

**CORRESPONDING AUTHORS:** Weimin Li, Email: [liweimin01@caas.cn](mailto:liweimin01@caas.cn);

Jinguang Yang, Email: [yangjinguang@caas.cn](mailto:yangjinguang@caas.cn).

**Supplementary Table 1.** Oligonucleotides used in this study^1^

| Oligo name | Organism | Application | Direction | Primer sequence (5'-3') |
| --- | --- | --- | --- | --- |
| PA_18_  P1  P2  TMV-5372-94 (Outer)  TMV-6023-44 (Inner)  ORSV-6065-85 (Outer)  ORSV-6460-80 (Inner)  CMV1-2698-717 (Outer)  CMV1-3015-34 (Inner)  CMV2-2441-60 (Outer)  CMV2-2723-42 (Inner)  CMV3-1628-47 (Outer)  CMV3-1867-86 (Inner)  TRV1-6177-97 (Outer)  TRV1-6419-39 (Inner)  TRV2-3363-82 (Outer)  TRV2-3550-70 (Inner)  TCV-3564-84 (Outer)  TCV-3937-57 (Inner)  PVX-5866-86 (Outer)  PVX-6045-65 (Inner)  PVY-9050-70 (Outer)  PVY-9334-54 (Inner)  PRRSV-15050-70 (Outer)  PRRSV-15119-39 (Inner)  PEDV-27596-616 (Outer)  PEDV-27721-741 (Inner)  FgHV2- 2428-48 (Outer)  FgHV2-12604-23 (Inner)  H1N1-7-772 -791 (Outer)  H1N1-7-814-835 (Inner)  H1N1-8-612-632 (Outer)  H1N1-8-659-680 (Inner)  RSV2-3211-31 (Outer)  RSV2-3260-80 (Inner)  RSV3-2153-73 (Outer)  RSV3-2203-23 (Inner)  RSV4-1774-94 (Outer)  RSV4-1812-33 (Inner)  RDV11(+)-599-620 (Outer)  RDV11(+)-727-48 (Inner)  RDV11(-)-376-56 (Outer)  RDV11(-)-306-286 (Inner)  RDV12(+)-681-701 (Outer)  RDV12(+)-771-91 (Inner)  RDV12(-)-334-14 (Outer)  RDV12(-)-299-79 (Inner)  AlRV(+)-3121-41 (Outer)  AlRV(+)-3230-49 (Inner) | ---  ---  ---  TMV  TMV  ORSV  ORSV  CMV (RNA1)  CMV (RNA1)  CMV (RNA2)  CMV (RNA2)  CMV (RNA3)  CMV (RNA3)  TRV (RNA1)  TRV (RNA1)  TRV (RNA2)  TRV (RNA2)  TCV  TCV  PVX  PVX  PVY  PVY  PRRSV  PRRSV  PEDV  PEDV  FgHV2  FgHV2  H1N1 (Segment 7)  H1N1 (Segment 7)  H1N1 (Segment 8)  H1N1 (Segment 8)  RSV (RNA2)  RSV (RNA2)  RSV (RNA3)  RSV (RNA3)  RSV (RNA4)  RSV (RNA4)  RDV (Segment 11)  RDV (Segment 11)  RDV (Segment 11)  RDV (Segment 11)  RDV (Segment 12)  RDV (Segment 12)  RDV (Segment 12)  RDV (Segment 12)  AlRV  AlRV | RT  Nested PCR  Nested PCR  Nested PCR  Nested PCR  Nested PCR  Nested PCR  Nested PCR  Nested PCR  Nested PCR  Nested PCR  Nested PCR  Nested PCR  Nested PCR  Nested PCR  Nested PCR  Nested PCR  Nested PCR  Nested PCR  Nested PCR  Nested PCR  Nested PCR  Nested PCR  Nested PCR  Nested PCR  Nested PCR  Nested PCR  Nested PCR  Nested PCR  Nested PCR  Nested PCR  Nested PCR  Nested PCR  Nested PCR  Nested PCR  Nested PCR  Nested PCR  Nested PCR  Nested PCR  Nested PCR  Nested PCR  Nested PCR  Nested PCR  Nested PCR  Nested PCR  Nested PCR  Nested PCR  Nested PCR  Nested PCR | ---  Reverse  Reverse  Forward  Forward  Forward  Forward  Forward  Forward  Forward  Forward  Forward  Forward  Forward  Forward  Forward  Forward  Forward  Forward  Forward  Forward  Forward  Forward  Forward  Forward  Forward  Forward  Forward  Forward  Forward  Forward  Forward  Forward  Forward  Forward  Forward  Forward  Forward  Forward  Forward  Forward  Forward  Forward  Forward  Forward  Forward  Forward  Forward  Forward | GCTGTCAACGATACGCTACGTAACGGCATGACAGTGA18  GCTGTCAACGATACGCTACGT  CTACGTAACGGCATGACAGTG  TGGAGTTTGTGTCGGTGTGTATT  GACTGCCGAAACGTTAGATGCT  CAACTCGTAGAGTTGATGATG  CGTGGTGCATACGATAATGCA  CACGAAATGGGTTTCTCAAT  GCTACGAGTACTGTGGTGTA  TGACAAACGTCGAACTCCAA  GTAACGAATGGGCGGAAGGT  AGTCCGTAAAGTTCCTGCCT  ACATCGAGCACCAACGCATT  GGTCATGCTAACAAATTGCGA  GTCTCATAATTCGAAGACCTC  TGGAACAAACCAGGAGCCAA  TCTCGACTGATCTTGATTGAT  GAGCACGATTGTCATTTTCTC  AGACTGGAAAACTAGTGCTCT  ACAGACACTATGGCACAGGC  AGTGGTATGGAACTGGATGT  ACATTTCTCAGATGTTGCAGA  CAAGTATGCATACTCTACTTG  TCCTCTAGCGACTGAAGATGA  GATCCAGACTGCCTTTAATCA  CAGCTGTTGATGGTGGTGATA  CTGAGTGTTTTTCTAGCGACT  CTAATCCTAGACCAACGAACA  AGGGACCAGAGTTCTTACCA  TCTACGCTGCAGTCCTCGCT  CACAAATCCTAAAATCCCCTTA  GTACAGAGGCCATGGTCATTT  GAATCCGCTCCACTATCTGCT  GTGGCAGCTTGTAATCGTAGT  CACACTAGCCCTGTTGTAACA  GCCTCTCACATATCTCATGGT  TGTCACCACCTTTGTCCTTCA  ATGGGTGAGAGGTTGATGAAA  GTATGTTGAAAGTTGCTCTCCT  CAAATACGATGCTGATCCCTT  GATCATATCCTCTTCTCGATTA  GAGCTTTCCCCTGGGTTAACA  AAGTACCGCTCCTGTGTATGA  CTCGATCTACTGGTTATGAGT  CTGACTGCCTACTTAACCTAT  GCGTTCTGATTATAGTGAGCA  GGACTCGAAGTTTCCGGTTAT  GACTATTGCGGGAATAGACA  CTTCTACCCTCCCGTGCTCT |

**^1^**The oligonucleotides for detection or isolation of viral sequences were designed from reference genomes of TMV (#NC_001367), ORSV (#X82130), CMV RNA1 (#D00356), CMV RNA2 (#D00355), CMV RNA3 (#D10538), TRV RNA1 (#AF166084), TRV RNA2 (#Z36974), TCV (#M22445), PVX (#EU571480), PVY（#JN083842）, PRRSV (#EU360130), PEDV (#JN825712), FgHV2 (KP208178), H1N1 S7 (NC_002016), H1N1 S8 (NC_002020), RSV RNA2 (JQ927428), RSV RNA3 (JQ927422), RSV RNA4 (JQ927416), RDV S11 (NC_003767), RDV S12 (NC_003768) and AlRV (KJ817371).
